# Argonaute 2 Complexes Selectively Protect the Circulating MicroRNAs in Cell-Secreted Microvesicles

**DOI:** 10.1371/journal.pone.0046957

**Published:** 2012-10-15

**Authors:** Limin Li, Dihan Zhu, Lei Huang, Jing Zhang, Zhen Bian, Xi Chen, Yuan Liu, Chen-Yu Zhang, Ke Zen

**Affiliations:** 1 Jiangsu Engineering Research Center for MicroRNA Biology and Biotechnology, State Key Laboratory of Pharmaceutical Biotechnology, School of Life Sciences, Nanjing University, Nanjing, Jiangsu, China; 2 CMBP, Department of Biology, Georgia State University, Atlanta, Georgia, United States of America; Cincinnati Children's Hospital Medical Center, United States of America

## Abstract

Cell-secreted miRNAs are highly stable and can serve as biomarkers for various diseases and signaling molecules in intercellular communication. The mechanism underlying the stability of circulating miRNAs, however, remains incompletely understood. Here we show that Argonaute 2 (Ago2) complexes and microvesicles (MVs) provide specific and non-specific protection for miRNA in cell-secreted MVs, respectively. First, the resistance of MV-encapsulated miRNAs to RNaseA was both depended on intact vesicular structure of MVs and protease-sensitive. Second, an immunoprecipitation assay using a probe complementary to human miR-16, a miRNA primarily located in the MVs and showed a strong, protease-sensitive resistance to RNaseA, identified Ago2 as a major miR-16-associated protein. Compared with protein-free miR-16, Ago2-associated miR-16 was resistant to RNaseA in a dose- and time-dependent fashion. Third, when the miR-16/Ago2 complex was disrupted by trypaflavine, the resistance of miR-16 to RNaseA was decreased. In contrast, when the association of miR-16 with the Ago2 complexes was increased during cell apoptosis, although the total amount of miR-16 and Ago2 remained unchanged, the resistance of miR-16 to RNaseA in the MVs was enhanced. A similar correlation between the increase of miR-223/Ago2 association and the resistance of miR-223 against RNaseA was observed during all *trans* retinoic acid (ATRA)-induced cell differentiation of promyelocytic HL60 cells. In conclusion, the association of miRNAs with Ago2 complexes, an event that is linked to cell functional status, plays a critical role in stabilizing the circulating miRNAs in cell-secreted MVs.

## Introduction

MicroRNAs (miRNAs) are a class of ∼22-nt-long, non-coding RNAs that negatively regulate the expression of target mRNAs [1], [2]. MiRNAs have been shown to be involved in the regulation of most biological processes, including differentiation, proliferation, apoptosis, and migration, and to be implicated in several diseases including cancer [2]–[4]. Recently, studies by our laboratory [5], [6] and others [7]–[10] have detected significant amounts of miRNA in extracellular human body fluids, including blood plasma, serum, urine, saliva and semen. More importantly, the unique expression patterns of circulating miRNAs in the blood have been successfully revealed to be biomarkers for various types of cancer, cardiovascular disease and organ injury [5]–[7], [11]–[13]. The secretion of miRNAs into the extracellular medium by mammalian cells in culture through either the exosomal pathway [11], [14]–[17] or an exosome-independent pathway [18], [19] has also been reported. However, the molecular basis underlying the high stability of the circulating miRNAs, particularly the circulating miRNAs that are in MVs that have been secreted from the original cells, remains largely unknown. It is widely believed that microvesicles (MVs) provide a general protection for circulating miRNAs, but certain circulating miRNAs are still resistant to RNase A after the disruption of the MVs, suggesting that these circulating miRNAs are stabilized by factors other than MVs. Recent studies by Arroyo et al. [19] and Turchinovich et al. [18] showed that the MV-free miRNAs were associated with Ago2, a major component of the RNA-induced silencing complex [20], [21], and were protected from RNaseA by the Ago2 complexes. However, the protective effect of Ago2 complexes or other proteins on the secreted miRNAs in the MVs has not been clearly defined.

In this study, we demonstrated that, in healthy human plasma and in culture medium from HeLa cells, the majority of the secreted miRNAs were located in cell-secreted MVs, and these MV-encapsulated miRNAs were bound to Ago2 complexes at various degrees. Both the vesicular structure of the MVs and the Ago2 complexes contribute to the high stability of the miRNAs in the MVs. Besides the general protection by MVs, the resistance of miRNAs in the MVs against RNase was also positively correlated with the degree of their association with Ago2 complexes. Furthermore, we found that the association of the secreted miRNAs with the Ago2 complexes was dependent on a particular cellular functional status and that the disruption or enhancement of the miRNA-Ago2 association in the MVs respectively decreases or increases the resistance of the miRNAs to RNaseA.

## Materials and Methods

### Reagents and antibodies

Trypaflavine (3,6-diamino-10-methylacridinium chloride, TPF) was purchased from Sigma-Aldrich (St Louis, MO). Synthetic RNA molecules, including pre-miR-16 and scrambled negative control oligonucleotides, were purchased from Ambion (Austin, TX, USA). Synthetic miR-16 and 3′- and 5′-biotin-labeled miR-16 oligonucleotides were purchased from Invitrogen (Shanghai, China). The mouse monoclonal anti-Ago2 (ab57113) and the rabbit polyclonal anti-Ago2 (ab32381) were purchased from Abcam (Hong Kong, China). The mouse monoclonal anti-GAPDH antibody and the Protein G-Agarose (sc-2003) were purchased from Santa Cruz Biotechnology (Santa Cruz, CA). Normal mouse IgG was purchased from Millipore (Cat.12–371).

### MV isolation

MVs were isolated from the plasma of healthy donors and from cell-culture medium by differential centrifugation, as described in previous publications [15], [22], [23]. All of the healthy donors provided written consent, and ethics permission was obtained for the use of the plasma and serum samples. Briefly, the plasma and cell culture medium were sequentially centrifuged at 300×g (30 min), 1200×g (30 min) and 10,000×g to purify the supernatant. The supernatant was then centrifuged at 110,000×g for 70 min (all of the steps were performed at 4°C).

### Cell culture

Human HeLa and promyelocytic HL60 cells were purchased from the China Cell Culture Center (Shanghai, China). The cells were maintained at 37°C in a humidified 5% CO_2_ incubator in Dulbecco's modified Eagle medium (DMEM) (Gibco) that contained 10% fetal bovine serum (Gibco), 100 units/ml of penicillin, and 100 µg/ml of streptomycin. The HeLa cells were pretreated with 10 µM trypaflavine (TPF) for two days, and the MVs were harvested from the culture medium. In separate experiment, human promyelocytic HL60 cells were treated with all *trans* retinoic acid (ATRA) to induce leukocyte-like cell differentiation.

### RNA isolation and qRT-PCR of mRNA or mature miRNAs

The total cell RNA was extracted using Trizol reagent (Invitrogen). The RNA from human plasma, MVs, MV-free plasma and immunoprecipitation products was isolated using the miRNeasy Mini Kit (QIAGEN). The qRT-PCR was performed using TaqMan probes (Applied Biosystems, for mature miRNAs) or SYBR Green (Takara, for mRNA or pre-miRNA) [6], [13]. Briefly, the total RNA was reverse-transcribed to cDNA using AMV reverse transcriptase (Takara) and a stem-loop RT primer or Reverse primer (Applied Biosystems). The real-time PCR was performed on an Applied Biosystems 7900 Sequence Detection System (Applied Biosystems). All of the reactions, including the no-template controls, were run in triplicate. After the reactions, the C_T_ values were determined using fixed threshold settings.

### Immunoprecipitation and immunoblotting

Cells or MVs were lysed with lysis buffer (20 mM Tris-HCl, 150 mM NaCl, 0.5% Nonidet P-40, 2 mM EDTA, 0.5 mM DTT, 1 mM NaF, 1 mM PMSF and 1% Protease Inhibitor Cocktail from Sigma, pH 7.5) for 30 min on ice. The lysates were cleared by centrifugation (16,000×g) for 10 min at 4°C and then immunoprecipitated with mouse monoclonal anti-Ago2 antibody or mouse normal IgG followed by protein G-Agarose beads. After the elution from the beads, the RNA was prepared using a miRNeasy Mini Kit. The miRNAs that were associated with Ago2 were analyzed by qRT-PCR. A rabbit polyclonal anti-Ago2 antibody was used for the western blot analysis of Ago2. The normalization was performed by blotting the same samples with an antibody against GAPDH.

### MiRNA pull-down assay

The miRNA pull-down assay was performed as described previously [24], with a minor modification. Briefly, a DNA probe that was complementary to human mature miR-16 was synthesized, labeled with biotin at both the 5′ and 3′ ends and dissolved in 500 µl of wash/binding buffer (500 mM NaCl, 20 mM Tris-HCl and 1 mM EDTA, pH 7.5) at a final concentration of 8.0 pmol/µl. The probe was then incubated with streptavidin-coated magnetic beads (New England Biolabs, Cat. S1420S) according to the manufacturer's instructions [24]. The MV lysate was pretreated with DNase I (Takara) and then incubated with probe-coated magnetic beads at 37°C for 3 h. After washing 6 times with the wash/binding buffer, a magnet was applied to attract the beads/miR-16/Ago2 complex to the side of the tube. The pull-down product was further analyzed by SDS-PAGE and western blotting, using antibodies against Ago2, CD63 or other proteins, respectively. The sequences of the probes used were as follows: anti-miR-16 pull-down probe, ACGCCAATATTACGTGCTGCTAA; random probe, TGATGTCTAGCGCTTGGGCTTTG; anti-miR-223 pull-down probe, ATGGGGTATTTGACAAACTGACAA.

### Statistical analysis

All of the images of the western blots and QRT-PCR assays were representative of at least three independent experiments. The qRT-PCR was performed in triplicate. The data were presented as the means ± SD for three or more independent experiments. The differences were considered to be statistically significant at *p*<0.05, assessed using Student's *t* test (for paired samples).

## Results

### Contribution of both MVs and proteins to the stability of circulating miRNAs in MVs

**Figure 1:**
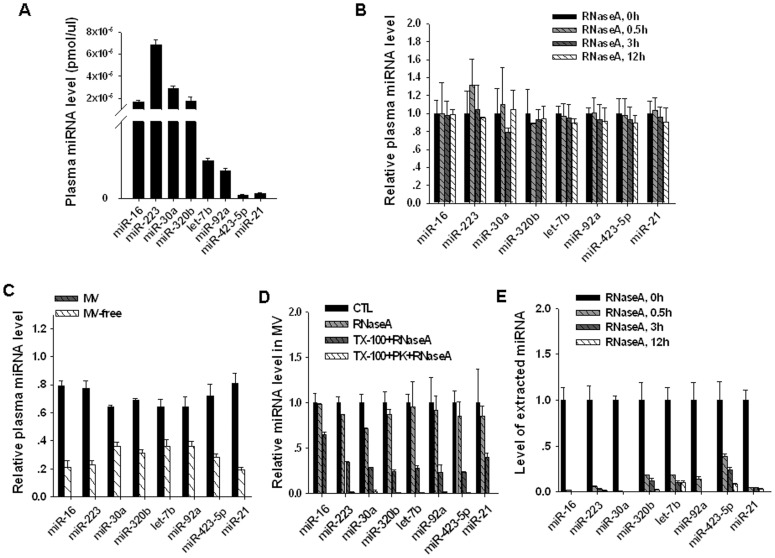
Both the vesicular structure of MVs and proteins contribute to the stability of secreted miRNAs in the plasma. A) The absolute levels of eight miRNAs in healthy human plasma detected by qRT-PCR. The concentration of the miRNA is calculated by referring to the standard curve. B) The resistance of plasma miRNAs to degradation by RNase A. Plasma (250 µl) was digested with 20 µg/ml RNase A for 0, 0.5, 3 and 12 h, respectively. C) The relative levels of miR-16, miR-223, miR-30a, miR-320b, let-7b, miR-92a, miR-423-5p and miR-21 in the MV vs. MV-free fractions of human plasma. D) The MV fractions (500 µg protein) were treated in the following three ways: 20 µg/ml RNase A for 3 h at 37°C; 0.1% Triton X-100 (TX-100) for 5 min and then 20 µg/ml RNase A for 3 h at 37°C; 0.1% TX-100 for 5 min, then 100 µg/ml PK for 2 h, followed by inactivation at 95°C for 15 min, and then 20 µg/ml RNase A for 3 h at 37°C. E) The degradation of the miRNAs extracted from human plasma (250 µl) by 20 µg/ml RNase A for 0, 0.5, 3 and 12 h, respectively.

Similar to circulating miRNAs in other body fluids, miRNAs in human plasma showed a significant stability against RNases and other harsh conditions, such as extreme temperature and pH [5], [25]. To characterize the stability of secreted miRNAs in plasma, we selected eight plasma miRNAs with relatively high copy numbers detected by Solexa sequencing (Table S1). Employing a TaqMan probe-based quantitative real time polymerase chain reaction (qRT-PCR) assay [6], [26], we first measured the levels of these miRNAs in the plasma of healthy donors. As shown in Figure 1A, miR-16, miR-223, miR-30a, miR-320b, let-7b, miR-92a, miR-423-5p and miR-21 were confirmed by qRT-PCR to have high expression levels in human plasma. When the human plasma was treated with 20 µg/ml RNaseA for various lengths of time, these miRNAs proved to be quite stable (Figure 1B). Interestingly, after separating the plasma into two fractions, MV and MV-free fractions, by sequential centrifugation, we found that these miRNAs were primarily localized in the MV fraction (Figure 1C). In this study, we selected these miRNAs to characterize the mechanisms underlying the stability of the circulating miRNAs in MVs. Because the majority of miRNAs tested in this study were found in the MVs, these circulating miRNAs might be generally protected by the vesicular structure of the MVs from the extracellular RNaseA. First, to test whether the MVs protects the miRNAs from degradation by RNaseA, we disrupted the MVs by adding 0.1% Triton X-100 (TX-100) during the RNaseA treatment. As shown in Figure 1D, the levels of these eight miRNAs were significantly decreased, indicating that the vesicular structure of the MVs does provide general protection for the miRNAs that are stored inside the MVs. However, even without intact MVs, these eight miRNAs still showed varying degrees of resistance to RNaseA (Figure 1D). For miR-16, more than 60% of the total miR-16 was intact and detected by a qRT-PCR assay after the disruption of the MVs, whereas less than 30% of the total miR-30a was detected. Second, to test whether proteins play a role in protecting the secreted miRNAs that are stored in MVs, we further added proteinase K (PK) into the digestion system containing TX-100 and RNaseA. As shown, all of the miRNAs were now completely degraded (Figure 1D). Together, these results suggest that both the MVs and proteins contribute to the resistance to RNase A of secreted miRNAs in the MVs. The protection by the MVs is non-specific, whereas the protection by proteins is selective for particular miRNAs. In agreement with the observation that the stability of secreted miRNAs is dependent on MVs and proteins, we found that all eight miRNAs, after extracted from the plasma, were completely degraded by RNaseA (Figure 1E), which is in agreement with the previous finding that the circulating miRNAs are generally not intrinsically resistant to RNases [19].

### Identification of Ago2 as a key protein protecting secreted miRNAs in MVs

**Figure 2:**
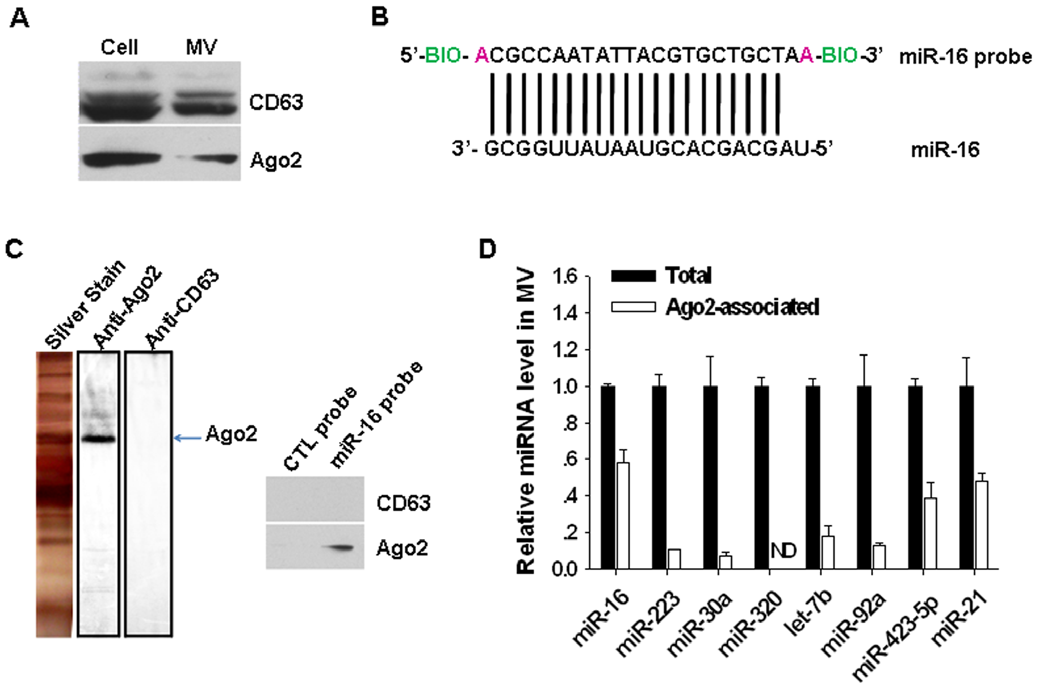
The identification of Ago2 as a key protein that associates with miRNAs in MVs. A) The localization of Ago2 and CD63 in both HeLa cells and HeLa MVs. B) A schematic illustration of the miR-16 pull-down strategy using a biotin-labeled probe complementary to human miR-16. C) Silver staining and western blotting of pull-down product from human plasma MVs by miR-16 probe. Note that, although both CD63 and Ago2 are expressed in MVs, only Ago2 is associated with miR-16. D) The percentage of individual miRNAs that are associated with Ago2 complexes in the MVs isolated from human plasma. ND, not detected.

Previous studies [11], [27]–[29] showed that Ago2 and CD63 were located in MVs. Employing CD63 as an exosomal marker, we confirmed that the isolated MVs from cultured HeLa cells were enriched in both CD63 and Ago2 (Figure 2A). Because the miR-16 in the MVs was strongly protected by a proteinase-sensitive mechanism (Figure 1D), we designed a miR-16 pull-down strategy to isolate potential miR-16-associated proteins using the MV fractions isolated from human plasma (Figure 2B). The pull-down product by the biotin-labeled probe complementary to human miR-16 (add adenosine at the 5′ and 3′ ends, respectively) was further separated by SDS-PAGE followed by silver staining or by western blotting using anti-Ago2 and anti-CD63 antibodies in a parallel fashion. As shown in Figure 2C, although both Ago2 and CD63 were enriched in the MVs, only Ago2 was found to be associated with miR-16. We also employed the same strategy to isolate potential miR-223-associated proteins using the MV fractions derived from human plasma (Figure S1). As can be seen, Ago2 was also identified as a major protein band associated with miR-223 though the amount of Ago2 associated with miR-223 was slightly less than that associated with miR-16. We then analyzed the association of various miRNAs with the Ago2 complexes in the MVs by immunoprecipitating Ago2 using an anti-Ago2 antibody, followed by the detection of the miRNAs using TaqMan probe-based qRT-PCR. Interestingly, we found that the association of the MV-encapsulated miRNAs with the Ago2 complexes was variable, and among the eight miRNAs that we tested, miR-16 showed the highest percentage of total miRNA to associate with the Ago2 complexes. Combining this observation with the result from Figure 1D, we speculate that the degree of association of miRNAs with the Ago2 complexes is positively correlated with the resistance of the miRNAs to degradation by RNaseA.

**Figure 3:**
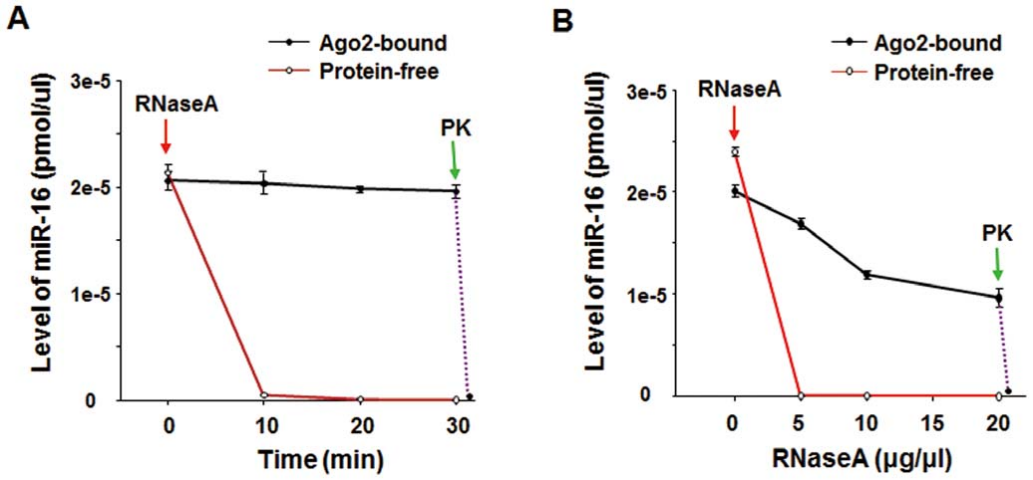
Ago2-associated miR-16 is highly resistant to RNaseA. A) Equal amounts of Ago2-associated miR-16 and protein-free, synthetic, mature miR-16 were treated with 20 µg/ml RNase A or 20 µg/ml RNaseA plus 100 µg/ml PK for various lengths of time. The Ago2 complex-associated miR-16 was obtained by immunoprecipitation using an anti-Ago2 antibody. B) Equal amounts of Ago2-associated miR-16 and protein-free, synthetic, mature miR-16 were treated with various concentrations of RNaseA or RNaseA plus 100 µg/ml PK for 30 min.

To further analyze the protection provided by Ago2 complexes to miRNAs in the MVs, we compared the stability of mature miR-16 that was associated with Ago2 complexes with that of free, synthetic, mature miR-16. In this experiment, the Ago2-associated miRNAs in the MVs, including miR-16, were harvested by immunoprecipitating the lysate of MV fraction using an anti-Ago2 antibody, and the amount of Ago2-associated miR-16 in the precipitated product was quantitatively analyzed by qRT-PCR, referring to the standard curve of miR-16. An equal amount of Ago2-associated miR-16 and free, synthetic miR-16 were then treated with RNaseA at various concentrations and for various time periods. As shown in Figure 3A and 3B, Ago2 complexes strongly protect miR-16 against RNaseA degradation in a time- and dose-dependent fashion, and the protection by the Ago2 complexes can be completely abolished by PK treatment.

**Figure 4:**
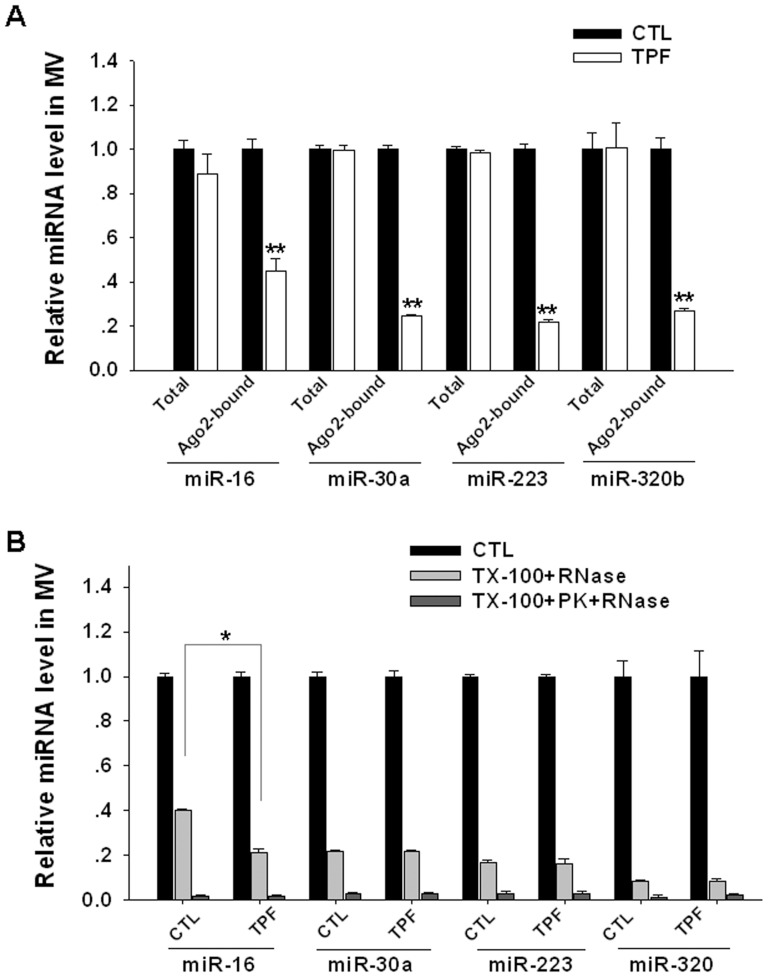
Decrease of the stability of the miRNAs in MVs by disrupting the association of the miRNA with Ago2 complexes. A) HeLa cells were treated with or without 8 µM TPF for 2 days and the MVs were collected from the culture supernatant. The levels of total miR-16, miR-30a, miR-223, miR-320b and Ago2 complex-associated miR-16, miR-30a, miR-223, miR-320b in the MVs were assessed by qRT-PCR. B) The resistance of miR-16, miR-30a, miR-223 and miR-320b in MVs with/without TPF treatment against RNaseA. The degradation assay of MV-encapsulated miRNAs was performed as the following two ways: treatment with a) 0.1%Triton X-100 (TX-100) for 5 min and then 20 µg/ml RNaseA for 30 min at 37°C, or b) 0.1% TX-100 for 5 min, then 100 µg/ml proteinase K (PK) for 2 h, followed by 95°C inactivated for 15 min, and then 20 µg/ml RNaseA for 30 min. *, p<0.05; **, p<0.01.

Recently, a small molecule named trypaflavine (TPF) has been discovered to block the loading of miRNAs into Ago2 complexes, possibly through disruption of the protein-protein association between TRBP and Ago2 [30]. We tested whether TPF treatment can decrease the stability of miRNAs, including miR-16, miR-30a, miR-223 and miR-320b, in secreted MVs by decreasing the miRNA-Ago2 association. In this experiment, HeLa cells were treated with or without 8 µM TPF for two days. The MVs were collected from the culture media and then used for an Ago2 pull-down assay. As shown in Figure 4A, we found no change in the total amount of each miRNA in the MVs, but the percentage of Ago2 complex-associated miRNAs was significantly reduced. This decrease of not the total miRNA level but the level of miRNA associated with Ago2 was also observed in HeLa cells treated with TPF (Figure S2B). Interestingly, the level of Ago2 in HeLa cells was not altered by TPF treatment (Figure S2A). As expected, the stability of miR-16 in the MVs derived from the TPF-treated HeLa cells was significantly lower than that of non-treated MVs (Figure 4B).

**Figure 5:**
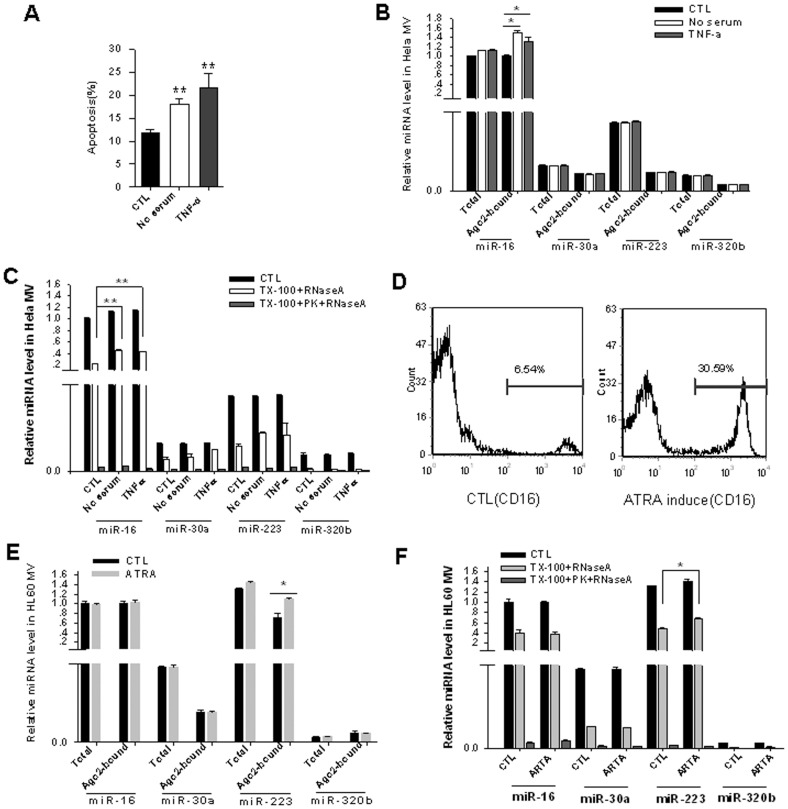
Enhancement of both the association of miRNAs with Ago2 complexes in cell-derived MVs and the resistance of miRNAs to RNaseA under various physiological conditions. A) The apoptosis of HeLa cells induced by serum starvation and TNFα treatment for 24 h, respectively. B) Relative levels of total and Ago2 complex-associated miR-16, miR-30a, miR-223 and miR-320b in the MVs derived from HeLa cells with or without apoptotic reagent treatment. Note that, although the total miR-16 levels are not changed, the percentage of miR-16 associated with Ago2 complexes is increased under apoptosis induced by either serum starvation or TNFα treatment. C) The resistance of miR-16 in HeLa cell-derived MVs to RNaseA. D) The differentiation of HL60 cells induced by 20 µM ATRA for 48 h. E) Relative levels of total miR-16, miR-30a, miR-223, miR-320b and the levels of these miRNAs associated with Ago2 complexes in the MVs derived from HL60 cells with or without induced by ATRA. Note that, although the total miR-223 levels are not changed, the percentage of miR-223 associated with Ago2 complexes is increased during ATRA-induced differentiation. F) The resistance of miR-223 in HL60 cell-derived MVs to RNaseA. *, p<0.05; **, p<0.01.

It has been shown that miR-16 [31] and miR-223 [32], [33] are linked to cellular apoptosis and differentiation process, respectively. Our previous study also showed that the intracellular distribution of miRNAs may be related to certain cellular functional states [24]. To study whether the association of MV-encapsulated miRNAs with Ago2 complexes and their resistance to RNaseA degradation is dynamically regulated by cellular biological function, we assessed the relationship between the association of Ago2 complexes with miR-16 or miR-223 and the resistance of these miRNAs to RNaseA under cell apoptotic or differentiation conditions. In these experiments, HeLa cells were treated with tumor necrosis factor α (TNFα) or serum-depleted cultured medium to induce apoptosis, while promyelocytic HL60 cells were treated with ATRA to induce cell differentiation [34]. The percentage of apoptotic HeLa cells was increased under both serum deprivation and TNFα treatment (Figure 5A). The MVs released by the HeLa cells were then collected from the culture medium for stability analysis. As shown in Figure 5B, under the early cell apoptotic conditions induced by serum depletion or TNFα, the percentage of miR-16 associated with Ago2 complexes in the MVs was markedly increased, although the total amount of miR-16 was not changed. A similar elevation of Ago2 complex-associated miR-16 but not total miR-16 was also observed in apoptotic HeLa cells (Figure S3A, lower panel). We also tested the total amount of cellular Ago2 under normal and apoptotic conditions and found no enhancement of the Ago2 expression level by apoptosis (Figure S3A, upper panel). As expected, with the percentage of Ago2-associated miR-16 being increased, the resistance of the miR-16 in the MVs to RNaseA was significantly enhanced (Figure 5C). TNFα treatment of HeLa cells also caused alteration of many miRNAs at cellular level. For example, the level of miR-483-5p in HeLa cells was upregulated by TNFα treatment (Figure S3, lower panel). We also tested the level of miR-483-5p and its association with Ago2 in MVs, and the data indicated that the levels of miR-483-5p associated with or without Ago2 in MVs were increased (Figure S4A). With the increased percentage of Ago2-associated miR-483-5p, the resistance of the miR-483-5p in the MVs to RNaseA was accordingly increased (Figure S4B). In a similar fashion, HL60 cells treated with ATRA expressed higher level of CD16, indicating a successful differentiation of promyelocytic HL60 cells to mature leukocyte (Figure 5D). During cell differentiation, the percentage of miR-223 associated with Ago2 complexes in the MVs was specifically increased, although the total amount of miR-223 was not altered (Figure 5E). In agree with the conclusion that Ago2 complex protects its associated miRNA, the resistance of the miR-223 in the MVs to RNaseA was significantly enhanced following ATRA-induced cell differentiation (Figure 5F). We also observed an elevation of Ago2 complex-associated miR-223 but not total miR-223 in ATRA-treated HL60 cells (Figure S3B, lower panel). In addition, the total amount of cellular Ago2 with or without ATRA treatment showed no alteration (Figure S3B, upper panel).

## Discussion

In this work, we addressed a key issue in the field of miRNA research: the molecular basis underlying the high stability of the circulating miRNAs in the cell-secreted MVs. Employing human peripheral blood and cell culture media as samples and TaqMan probe-based miRNA qRT-PCR assays as the main tool, we characterized the contributions of the vesicular structure of MVs and the association of miRNAs with Ago2 complexes to the resistance of miRNAs to RNases.

It has been suggested that circulating miRNAs can be derived from three pathways [35], [36]: a) active secretion by living cells via MVs, including exosomes and shedding vesicles [16], [25], a pathway that is tightly regulated by various factors and that may be a common avenue for cells reacting to various stimuli; b) active secretion from cells in a protein/miRNA complex fashion; and c) passive leakage from broken or damaged cells. In agreement with many previous studies [11], [16], [17], [37], [38], we found that the majority of the circulating miRNAs in human peripheral blood, such as miR-16, miR-223, miR-30a, miR-320, let-7b, miR-92a, miR-423-5p and miR-21 were located in the MV fraction. The vesicular structures of the exosomes not only provide a general protection against RNases, but also deliver the miRNAs into their target cells with high efficiency. However, recent studies also showed that the majority of circulating miRNAs, including miR-16, were not associated with cell-derived microvesicles [18], [19]. In addition, they found that these MV-free miRNAs were also associated with Ago2 complexes and thus were RNaseA-resistant. Based on their results, these Ago2-associated miRNAs in the MV-free plasma may be passively leaked from broken cells or directly released from living cells via a protein-mediated secretion pathway. However, there is no evidence for the Ago2-mediated direct secretion of miRNAs from living cells. The different results regarding the distribution of circulating miRNA inside or outside the MVs may be due to the differences in various experimental procedures. Sequential ultracentrifugation or cell fractionation assays might cause the breakage of miRNAs from the MVs. Nevertheless, our results did not exclude the possibility that certain circulating miRNAs may primarily exist in an MV-free form.

Besides the general protection provided by MVs, our data clearly indicate that secreted miRNAs in MVs are protected by Ago2 complexes to various degrees. Interestingly, we found that not all of the miRNAs in the MVs were associated with the Ago2 complexes, and different miRNAs were associated with the Ago2 complexes to different degrees. Therefore, the protection provided by Ago2 complexes to the various miRNAs in the MVs was different. This finding may raise an important question about the fate of the circulating miRNAs in the cell-derived MVs. Because Ago2 is also a key effector of miRNA function, our results may imply that only the secreted miRNAs that are associated with Ago2 complexes in the cell-derived MVs are stable and have biological function after they enter into the recipient cells, whereas the non-Ago2 complex-bound miRNAs in the MVs may be simply degraded in the recipient cells. Our results further showed that the stability of a circulating miRNA in the cell-derived MVs is positively correlated with the degree of its association with Ago2 complexes. As shown in the *in vitro* digestion assay with RNase A (Figure 3), Ago2 complex-associated miR-16 was significantly resistant to RNase A compared with free miR-16, which was rapidly degraded by RNase A. These results suggest that certain cell secreted miRNAs are pre-loaded with Ago2 complexes in MVs released by origin cells and can be delivered into recipient cells where they start inhibiting their targets. In other words, the secreted miRNAs in MVs are already functionally equipped with Ago2 and can directly execute their roles in the recipient cells. Therefore, MV-delivery of secreted miRNAs provides a new mechanism for cell-to-cell communication. The Ago2/miRNA complexes are also highly protease-resistant, as miRNA remained stable in the cell lysates for over a week (data not shown). The unusual stability of the circulating miRNAs, particularly the miRNAs in cell-derived MVs, provides a solid grounding for the circulating miRNAs to serve as an ideal biomarker for various diseases and also as a novel class of signaling molecules in cell-cell communication.

Unlike other RNA species, circulating miRNA remains stable in the peripheral blood and culture medium for long periods due to the significant resistance of the nuclease to degradation. The specific role of Ago2 complexes in the stability of circulating miRNAs has been tested in the present study. Through the disruption of the association of miRNAs, including miR-16, with Ago2 complexes by TPF treatment, we successfully decreased the resistance of miRNAs in the cell-derived exosomes to RNase A. In contrast, when we increased the percentage of Ago2 complex-associated miR-16 by inducing apoptosis or the percentage of Ago2 complex-associated miR-223 by inducing cell differentiation, we found that the resistance of miR-16 or miR-223 in the cell-derived exosomes to RNase A was significantly enhanced. Interestingly, the total level of miRNAs shuttle by MVs seems not affected by the TPF's blockade of miRNA bound with Ago2, suggesting that miRNA sorting into the MVs may be not dependent on their binding capacity to Ago2.

Although our data showed that Ago2 complexes play a critical role in stabilizing secreted miRNAs in the MVs, it is necessary to mention that there are some discrepancies between miRNA association with Ago2 complexes and RNaseA protection by Ago2 complexes. For instance, although miR-16 and miR-223 differ in their protein mediated stability by about 50% (Figure 1D), the difference in Ago2 association of these two miRNAs is far greater (∼85%) (Figure 2D). The similar discrepancy was also observed in miR-320b, which is almost not associated with Ago2 complexes (Figure 2D) but still shows certain resistance to RNaseA (Figure 1D). Since protein digestion by PK dramatically enhances the sensitivity of miRNAs such as miR-223 and miR-320b to RNaseA although they are not associated with Ago2 at relatively high level, it is likely that these miRNAs in the cell-secreted MVs may be protected by other protein(s). In other words, Ago2 is not the only protein modulating the stability of extra cellular miRNAs.

In summary, our results collectively show that both the vesicular structure of the cell-derived MVs and the Ago2 complexes contribute to the stability of circulating miRNAs in the MVs. While the vesicular structure of MVs provides general protection to the MV-encapsulated miRNAs, the Ago2 complexes selectively associate with miRNAs in the MVs under certain cellular functional status and protect these cell-secreted miRNAs from degradation by RNases or proteases.

## Supporting Information

Table S1 **Plasma miRNA level detected by Solexa Sequencing.** Total miRNA copy number = 3780436. Only miRNAs with copy number ≥1500 were shown.
(DOCX)

Figure S1 **The identification of Ago2 as a key protein that associates with miRNAs in MVs.** A) A schematic illustration of the miR-223 pull-down strategy using a biotin-labeled probe complementary to human miR-223. B) Silver staining and Western blotting (WB) of pull-down product from human plasma MVs by miR-223 probe.
(DOC)

Figure S2 **Decrease of the stability of miRNAs in cell by disrupting the association of miRNA with Ago2 complexes.** A) HeLa cells were treated with or without 8 µM TPF for 2 days. The level of Ago2 is detected by western blotting. B) The levels of total miR-16, miR-30a, miR-223 and miR-320b, as well as Ago2 complex-associated miR-16, miR-30a, miR-223 and miR-320b in cells were assessed by qRT-PCR. **, p<0.01.
(DOC)

Figure S3 **Specific enhancement of the association of miRNAs with Ago2 complexes in cells under different physiological condition.** A) Upper panel, Ago2 expression level in HeLa cells induced by serum starvation and TNFα is detected by western bolting; Lower panel, relative levels of total miR-16, miR-30a, miR-223, miR-320b and miR-423-5p, as well as Ago2 complex-associated miR-16, miR-30a, miR-223, miR-320b and miR-423-5p in the HeLa cells with or without apoptotic reagent treatment. B) Upper panel, Ago2 expression level in HL60 cells induced by ATRA is detected by western blotting; Lower panel, relative levels of total miR-16, miR-30a, miR-223 and miR-320b, as well as Ago2 complex-associated miR-16, miR-30a, miR-223 and miR-320b in the HL60 cells with or without ATRA treatment. *, p<0.05; **, p<0.01.
(DOC)

Figure S4 **Enhancement of the association of miR-423-5p with Ago2 complexes in HeLa MVs by TNFα treatment.** A) Relative levels of total miR-423-5p, as well as Ago2 complex-associated miR-423-5p in the HeLa cell-derived MVs. Prior to MV isolation, HeLa cells were treated with or without TNFα. B) The resistance of miR-423-5p in HeLa cell-derived MVs to degradation by RNaseA. *, p<0.05; **, p<0.01.
(DOC)
