# Supplementary material for: Argonaute 2 Complexes Selectively Protect the Circulating MicroRNAs in Cell-Secreted Microvesicles
Source: PLoS One. 2012 Oct 15;7(10):e46957. doi: 10.1371/journal.pone.0046957 (PMC3471944; doi:10.1371/journal.pone.0046957)
Supplement: Table S1 — Plasma miRNA level detected by Solexa Sequencing. Total miRNA copy number = 3780436. Only miRNAs with copy number ≥1500 were shown. (DOCX) [file pone.0046957.s001.docx]

Table S1. Plasma miRNA level detected by Solexa Sequencing. Total miRNA copy number=3780436. Only miRNAs with copy number≥1500 were shown.

| miRNA | Copy Number | miRNA | Copy Number |
| --- | --- | --- | --- |
| hsa-let-7a | 404763 | hsa-miR-223 | 7637 |
| hsa-miR-320a | 343325 | hsa-let-7e | 7350 |
| hsa-let-7i | 334404 | hsa-miR-10a | 6677 |
| hsa-let-7b | 328414 | hsa-miR-744 | 6367 |
| hsa-let-7f | 314329 | hsa-miR-24 | 6007 |
| hsa-miR-423-5p | 236808 | hsa-miR-30d | 5842 |
| hsa-miR-103 | 224384 | hsa-miR-93 | 5406 |
| hsa-miR-122 | 205228 | hsa-miR-15b | 5295 |
| hsa-miR-107 | 161563 | hsa-miR-15a | 5291 |
| hsa-miR-140-3p | 102802 | hsa-miR-26b | 4786 |
| hsa-miR-21 | 86747 | hsa-miR-33a | 4311 |
| hsa-miR-92a | 80776 | hsa-miR-30e | 3960 |
| hsa-miR-101 | 78294 | hsa-miR-423-3p | 3730 |
| hsa-let-7g | 77591 | hsa-miR-148b | 3460 |
| hsa-miR-221 | 66377 | hsa-miR-128 | 3432 |
| hsa-miR-199a-3p | 63937 | hsa-miR-26a | 2736 |
| hsa-miR-199b-3p | 63937 | hsa-miR-143 | 2697 |
| hsa-miR-486-5p | 62456 | hsa-miR-1974 | 2637 |
| hsa-miR-451 | 59943 | hsa-miR-191 | 2534 |
| hsa-miR-185 | 56019 | hsa-miR-142-5p | 2348 |
| hsa-miR-25 | 37268 | hsa-miR-186 | 2339 |
| hsa-miR-320b | 37217 | hsa-miR-126* | 2297 |
| hsa-let-7d | 25199 | hsa-miR-146a | 2209 |
| hsa-miR-222 | 23503 | hsa-miR-502-3p | 2194 |
| hsa-miR-27a | 16024 | hsa-miR-660 | 2194 |
| hsa-miR-192 | 14902 | hsa-miR-629 | 2193 |
| hsa-miR-23a | 14456 | hsa-miR-500* | 2190 |
| hsa-miR-16 | 14349 | hsa-miR-1 | 2100 |
| hsa-miR-378 | 13625 | hsa-miR-320d | 2003 |
| hsa-let-7c | 9615 | hsa-miR-23b | 1876 |
| hsa-miR-29a | 9560 | hsa-miR-30a | 1857 |
| hsa-miR-27b | 8464 | hsa-miR-130a | 1727 |
| hsa-miR-22 | 8449 | hsa-miR-425* | 1720 |
| hsa-miR-320c | 7776 | hsa-miR-98 | 1713 |
