# Supplementary figures and images for: Argonaute 2 Complexes Selectively Protect the Circulating MicroRNAs in Cell-Secreted Microvesicles
Source: PLoS One. 2012 Oct 15;7(10):e46957. doi: 10.1371/journal.pone.0046957 (PMC3471944; doi:10.1371/journal.pone.0046957)

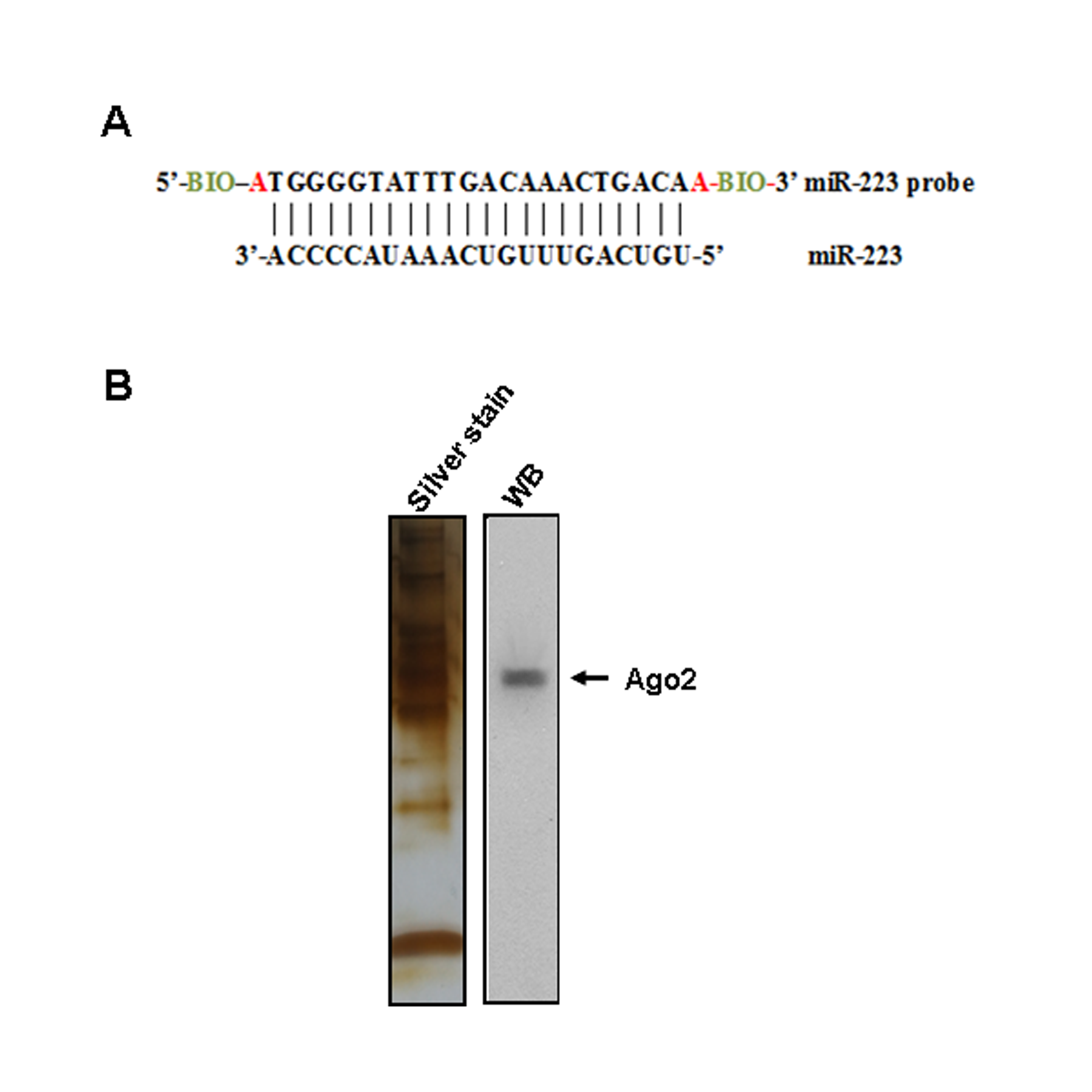


Figure S1

Supplement: Figure S1 — The identification of Ago2 as a key protein that associates with miRNAs in MVs. A) A schematic illustration of the miR-223 pull-down strategy using a biotin-labeled probe complementary to human miR-223. B) Silver staining and Western blotting (WB) of pull-down product from human plasma MVs by miR-223 probe. (DOC) [file pone.0046957.s002.doc]

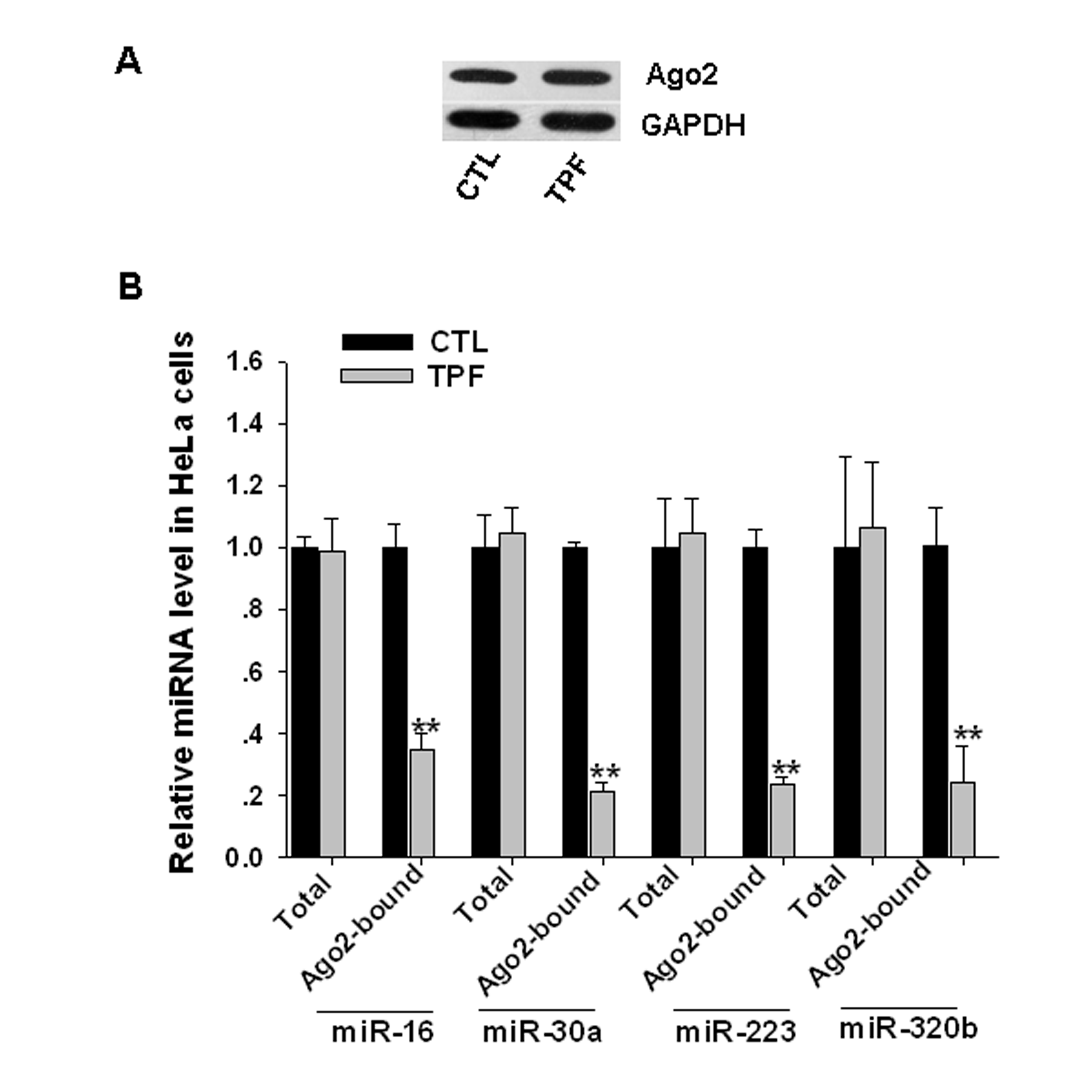


Figure S2

Supplement: Figure S2 — Decrease of the stability of miRNAs in cell by disrupting the association of miRNA with Ago2 complexes. A) HeLa cells were treated with or without 8 µM TPF for 2 days. The level of Ago2 is detected by western blotting. B) The levels of total miR-16, miR-30a, miR-223 and miR-320b, as well as Ago2 complex-associated miR-16, miR-30a, miR-223 and miR-320b in cells were assessed by qRT-PCR. **, p<0.01. (DOC) [file pone.0046957.s003.doc]

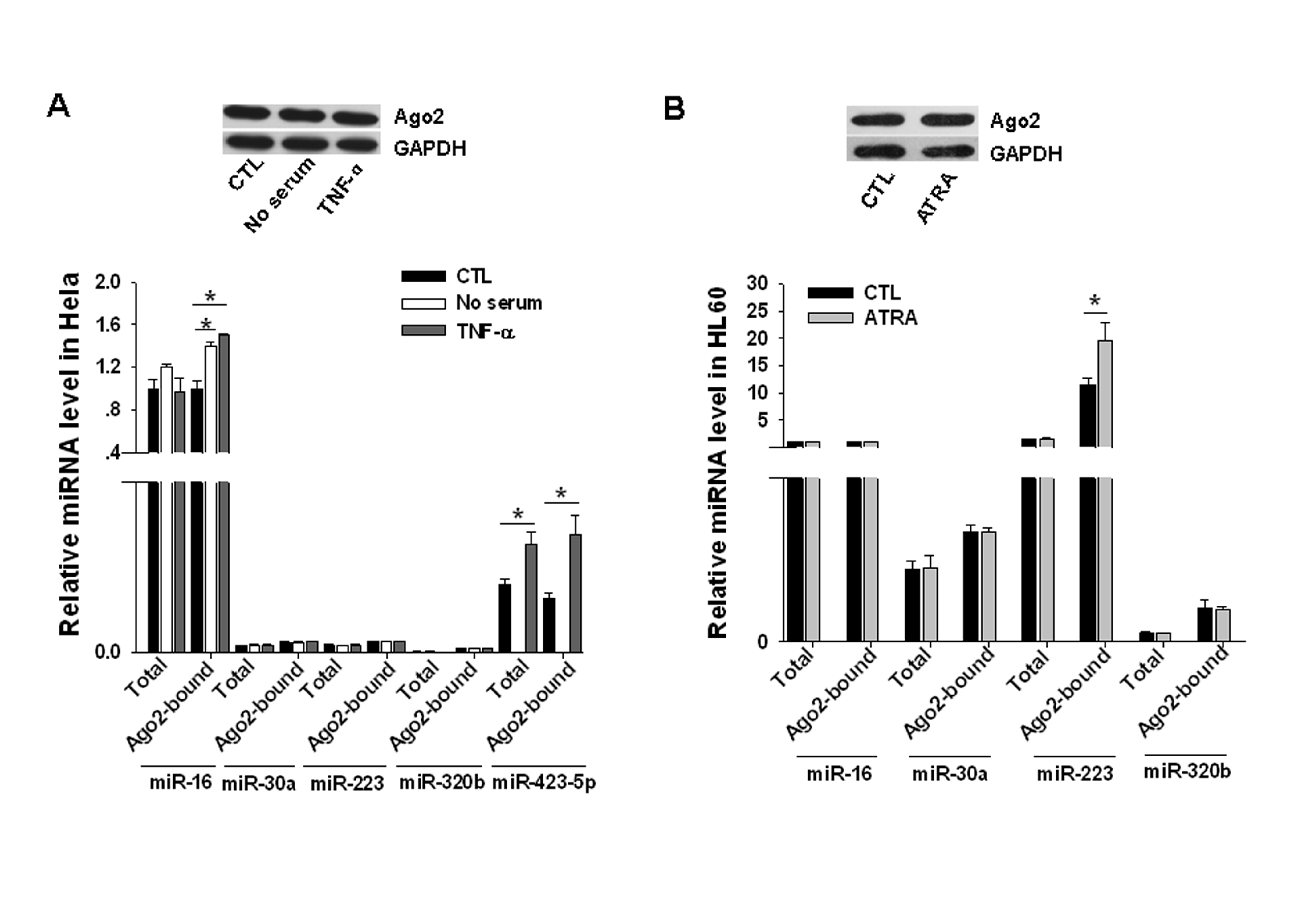


Figure S3

Supplement: Figure S3 — Specific enhancement of the association of miRNAs with Ago2 complexes in cells under different physiological condition. A) Upper panel, Ago2 expression level in HeLa cells induced by serum starvation and TNFα is detected by western bolting; Lower panel, relative levels of total miR-16, miR-30a, miR-223, miR-320b and miR-423-5p, as well as Ago2 complex-associated miR-16, miR-30a, miR-223, miR-320b and miR-423-5p in the HeLa cells with or without apoptotic reagent treatment. B) Upper panel, Ago2 expression level in HL60 cells induced by ATRA is detected by western blotting; Lower panel, relative levels of total miR-16, miR-30a, miR-223 and miR-320b, as well as Ago2 complex-associated miR-16, miR-30a, miR-223 and miR-320b in the HL60 cells with or without ATRA treatment. *, p<0.05; **, p<0.01. (DOC) [file pone.0046957.s004.doc]

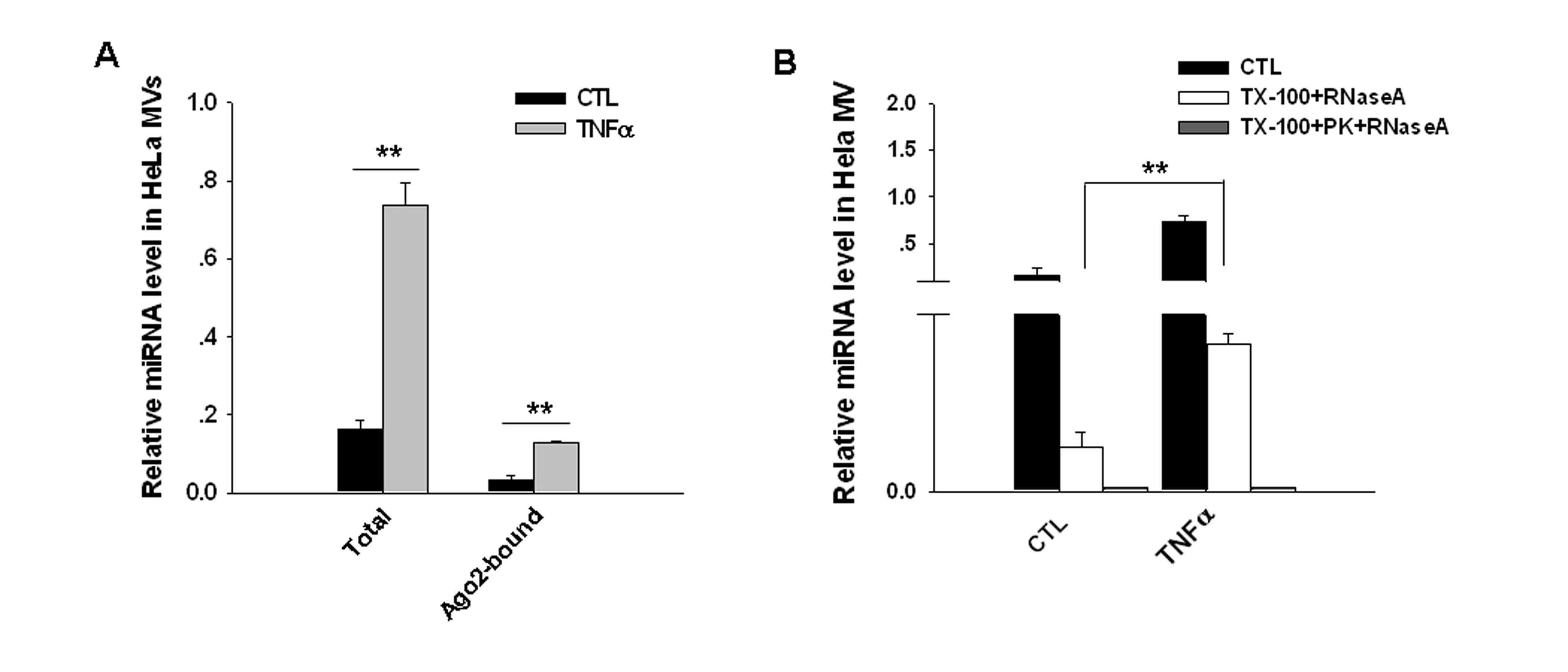


Figure S4

Supplement: Figure S4 — Enhancement of the association of miR-423-5p with Ago2 complexes in HeLa MVs by TNFα treatment. A) Relative levels of total miR-423-5p, as well as Ago2 complex-associated miR-423-5p in the HeLa cell-derived MVs. Prior to MV isolation, HeLa cells were treated with or without TNFα. B) The resistance of miR-423-5p in HeLa cell-derived MVs to degradation by RNaseA. *, p<0.05; **, p<0.01. (DOC) [file pone.0046957.s005.doc]
